# Novel Perspectives for Sensory Analysis Applied to Piperaceae and Aromatic Herbs: A Pilot Study

**DOI:** 10.3390/foods14010110

**Published:** 2025-01-03

**Authors:** Isabella Taglieri, Alessandro Tonacci, Guido Flamini, Pierina Díaz-Guerrero, Roberta Ascrizzi, Lorenzo Bachi, Giorgia Procissi, Lucia Billeci, Francesca Venturi

**Affiliations:** 1 Interdepartmental Research Centre “Nutraceuticals and Food for Health”, University of Pisa, Via del Borghetto 80, I-56124 Pisa, Italy; isabella.taglieri@unipi.it (I.T.); guido.flamini@unipi.it (G.F.); roberta.ascrizzi@unipi.it (R.A.); francesca.venturi@unipi.it (F.V.); 2 Department of Agriculture, Food and Environment, University of Pisa, Via del Borghetto 80, I-56124 Pisa, Italy; pierina.guerrero@phd.unipi.it; 3 Institute of Clinical Physiology, National Research Council of Italy (IFC-CNR), I-56124 Pisa, Italy; lorenzo.bachi@santannapisa.it (L.B.); giorgiaprocissi@gmail.com (G.P.); lucia.billeci@cnr.it (L.B.); 4 Department of Pharmacy, University of Pisa, Via Bonanno Pisano 6, I-56126 Pisa, Italy

**Keywords:** autonomic nervous system, aromatic herbs, EEG, electrocardiogram, emotions, GC-MS, sensory analysis, spices, VOCs, wearable sensors

## Abstract

Spices and aromatic herbs are important components of everyday nutrition in several countries and cultures, thanks to their capability to enhance the flavor of many dishes and convey significant emotional contributions by themselves. Indeed, spices as well as aromatic herbs are to be considered not only for their important values of antimicrobial agents or flavor enhancers everybody knows, but also, thanks to their olfactory and gustatory spectrum, as drivers to stimulate the consumers’ memories and, in a stronger way, emotions. Considering these unique characteristics, spices and aromatic herbs have caught the attention of consumer scientists and experts in sensory analysis for their evaluation using semi-quantitative approaches, with interesting evidence. In this pilot study as a first step, each studied botanical, belonging to Piperaceae or aromatic herbs, has been subjected to headspace solid phase micro-extraction (HS-SPME) coupled with gas-chromatography mass spectrometry (GC-MS) analysis to assess their spontaneous volatile emission, representing the complex chemical pattern, which encounters the consumers’ olfactory perception. Furthermore, the present investigation, performed on 12 individuals, outlines the administration of a pilot study, merging the typical sensory analysis with emotional data collection and the innovative contribution related to the study around the Autonomic and Central Nervous System activation in consumers, performed using wearable technologies and related signal processing. The results obtained by our study, beyond demonstrating the feasibility of the approach, confirmed, both in terms of emotional responses and biomedical signals, the significant emotional potential of spices and aromatic herbs, most of which featuring an overall positive valence, yet with inter-subjects’ variations. Future investigations should aim to increase the number of volunteers evaluated with such an approach to draw more stable conclusions and attempting a customization of product preferences based on both implicit and explicit sensory responses.

## 1. Introduction

For millennia, spices and aromatic herbs have been utilized globally to enhance and diversify culinary dishes, highlighting the unique characteristics of ethnic cuisines. For example, turmeric is integral to Indian cuisine, while basil, garlic, and oregano are prominent in Italian and Greek cuisines, and paprika powder is essential in Hungarian cuisine [1]. Unlike vegetables and fruits, which are primarily consumed for their macronutrient content, herbs and spices are used in minimal amounts to enhance the flavor, color, or presentation of foods. This is feasible thanks to the presence of specific chemical compounds that confer distinctive sensory properties [2,3]. These compounds include terpenes, alkaloids, flavonoids, phenolic compounds (such as polyphenols), and salicylates, among others [4,5].

Additionally, they contain several phenolic compounds that have been proven to provide antioxidative effects in food. Several studies have shown that spices and herbs such as rosemary, sage, and oregano, which are rich in bioactive compounds, act as powerful antioxidants. Therefore, they can be used to safely extend the shelf life of foods while enhancing their organoleptic profile and sensory features [6,7,8].

In addition, a recent proposal indicates that piperine, the primary trigeminal stimulant in black pepper, may modulate the perception of saltiness [9,10]. Therefore, black pepper, particularly its piperine component, could be viewed as a potential taste or flavor enhancer [11]. This implies that spices and herbs could be explored as possible strategies for reducing salt intake in human diets [12,13,14,15,16].

In summary, herbs and spices can serve three primary purposes: (1) as condiments, providing color and flavor to food while improving their shelf life; (2) as components in cosmetics and medicine; and (3) as natural dyes for cotton, silk, and wool [17,18].

Although comprehensive sensory data on spices and herbs are scarce in the literature, they can be classified by flavor into four categories: hot spices (i.e., black and white pepper, Cayenne pepper, mustard, chilies), mild-flavored spices (i.e., paprika, coriander), aromatic spices (i.e., clove, cumin, dill, fennel, nutmeg, mace, cinnamon), and aromatic herbs and vegetables (i.e., thyme, basil, bay leaf, marjoram, shallot, onion, garlic) [17].

On the other hand, from an evolutionary perspective, human senses are used to detect risks and adopt countermeasures, with a process that strongly depends not only on specific genetic characteristics and physiological conditions but also on cultural and social background. This fundamental associative learning process is key for guiding daily choices and its effectiveness is enhanced by the close association between olfactory (and to a lesser extent gustatory) perceptions and emotions realized at the thalamic level, making emotions not a result of sensory stimuli, but an integral part of perception [19,20].

Emotions can be defined as the responses triggered by the perception of relevant stimuli, encompassing physiological changes, cognitive responses, and may also be evoked by recalled or imagined events [21,22]. According to a growing body of science, emotions play a fundamental role in cognitive-sensory processing [23,24,25] during tasting of complex and iconic products such as wine and coffee [20,22,26,27].

Given the significant role spices and aromatic herbs play in various cultures, it is reasonable to assume that these botanicals may evoke a wide range of emotions during tasting experiences. However, to the best of our knowledge, no data on this topic is currently available in the literature. Bridging this gap would require developing a method to measure the emotions evoked in consumers by spices and aromatic herbs. The ultimate goal would be to identify the optimal selection of botanicals for seasoning, thereby enhancing the overall enjoyment of the final dish.

As previously reported by our group for complex red wines and coffee [20,26,27], in general, the expression of emotion can take place with two levels of central orders, closely related to each other in the manifestation of emotion itself: the classification, which is the excitation resulting from positive responses of the autonomic nervous system (ANS), and the affective response, which can be either negative or positive.

To this extent, in this study, an innovative method based on the synergy between both explicit (panel test assessed by trained judges) and implicit (biomedical signal-based) measurements was applied aiming at characterizing emotions aroused by a selection of spices and aromatic herbs. The applied multidisciplinary approach includes the following: (i) the characterization of the selected botanicals through descriptive sensory analysis, including both quantitative and hedonic parameters together with emotions aroused during tasting; (ii) the measure of emotions of trained judges aroused by botanicals through dedicated questionnaires focused on metaphorical language and emotions lexicon (explicit measurements); (iii) the evaluation of VOCs profile of botanicals via GC-MS in order to explore the possible correlations between VOCs composition, smell profile, and emotions aroused; (iv) the application of minimally obtrusive, wearable devices (e.g., commercial wearable sensors) to measure a range of biomedical signals of interest for research and clinical purposes, including electrocardiogram (ECG) and galvanic skin response (GSR) to match the Autonomic Nervous System (ANS) activation (implicit measurements). To complete the measure of physiological reactions during smelling, and in order to obtain information from the Central Nervous System (CNS), a subgroup of panelists was provided also with wearable devices for EEG tracking, with the aim to verify the feasibility of including this measure to the previously mentioned ones, in the framework of a whole multitasking approach.

Overall, to the best of our knowledge, this is the first time that such tools and analytical methods have been applied together to a panel test dealing with spices and aromatic herbs.

In this preliminary pilot study, we intentionally focused on the technological methodology to determine whether our approach could effectively detect changes elicited during tasting by different spices and/or aromatic herbs. However, we did not collect data on the judges’ prior psychological and psychophysical states, which may have influenced the final outcomes of the experiment. Accordingly, no data were collected about other possible bias related to dietary habits nor previous exposure of single judge to specific spice and aromatic herb.

## 2. Materials and Methods

### 2.1. Botanicals

Ten commercial botanicals provided by Italpepe2 S.r.l. (Italpepe2 S.r.l., Rome, Italy) selected among the most widespread Piperaceae and aromatic herbs diffused worldwide for dressing and cooking were used for the evaluation of their VOCs profile, sensory analysis and “emotional power”. The detailed list of samples, including sample code, type of botanical, scientific name, as well as general name, is shown in Table 1.

### 2.2. Chemical Analysis

#### 2.2.1. Headspace—Solid Phase Micro Extraction (HS-SPME)

Supelco (Merck KGaA, Darmstadt, Germany) SPME (Solid Phase Micro-Extraction) devices coated with Polydimethylsiloxane (PDMS) were used to sample the headspaces. SPME sampling was performed using the same new fiber, preconditioned according to the manufacturer instructions, for all the analyses. Sampling was accomplished in an air-conditioned room (22 ± 1 °C) to guarantee a stable temperature. After the equilibration time, the fiber was exposed to the headspace for a suitable amount of time based on the analyzed sample, experimentally determined to avoid under- and over-saturation of the fiber. In detail, sampling times varied from 10 s for Piper cubeba, Piper nigrum, Pimpinella anisum, Schinus molle, Pimenta dioica, to 20 s for Elettaria cardamomum, 60 s for Dipteryx odorata and 2 min for Piper longum.

No mixing or heating was applied during sampling. Once sampling was finished, the fiber was withdrawn into the needle and transferred to the injection port of the GC-MS system. The desorption conditions were identical for all the samples. Furthermore, blanks were performed before each first SPME extraction and randomly repeated during each series. Quantitative comparisons of relative peaks areas were performed between the same chemicals in the different samples.

#### 2.2.2. Gas Chromatography—Mass Spectrometry (GC-MS) Analysis

Gas chromatography–electron impact mass spectrometry (GC–EIMS) analyses were conducted with an Agilent 7890B gas chromatograph (Agilent Technologies Inc., Santa Clara, CA, USA) equipped with an Agilent HP-5MS (Agilent Technologies Inc., Santa Clara, CA, USA) capillary column (30 m × 0.25 mm; coating thickness 0.25 μm) and an Agilent 5977B single quadrupole mass detector (Agilent Technologies Inc., Santa Clara, CA, USA). Analytical conditions were as follows: injector and transfer line temperatures 220 and 240 °C, respectively; oven temperature programmed from 60 to 240 °C at 3 °C/min; carrier gas helium at 1 mL/min; split ratio 1:25. Acquisition parameters were as follows: full scan; scan range: 30–300 *m*/*z*; scan time: 1.0 s. The identification of the constituents was based on the comparison of their retention times with those of the authentic samples (when available), comparing their linear retention indices relative to the series of n-hydrocarbons (C6–C25). Computer matching was also used against a commercial [28] and a laboratory-developed mass spectra library built up from pure substances and components of commercial essential oils of known composition and MS literature data [29].

### 2.3. Sensory Analysis

Sensory analysis was conducted by a panel of 12 trained assessors (7 females and 5 males) aged 25 to 63 years. The assessors were selected based on their availability from a larger pool of judges who regularly collaborate with the Department of Agriculture, Food, and Environment (DAFE) at the University of Pisa. All judges underwent standardized training [30] to enhance their ability to recognize, describe, and quantify tastes, odors, and texture properties in accordance with ISO 8586 standards. All participants were primarily experts in the evaluation of foods and essential oils, and, before the start of the study, they had given their informed consent [31].

In each tasting session a sample was randomly presented twice to the judges to assess the panelist’s performance repeatability [32].

To minimize interference from sample handling, each blind-coded sample was presented to the judges in its own glass container, which was specifically covered with steel foil and opened just before the tasting session. Each sample was sniffed for 5 s, followed by a 1 min rest between consecutive evaluations.

**Figure 1:**
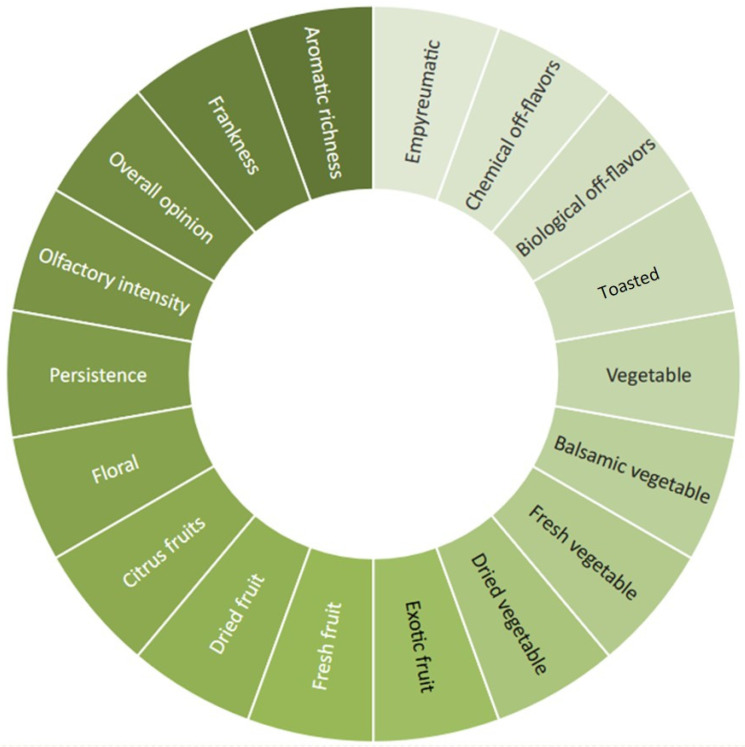
Olfactory wheel preliminary developed by the consensus panel.

The sensory analysis session was conducted at room temperature, in a standard sensory laboratory (ISO 8589:2010) [33]. The sensory profile was assessed through a detailed olfactory wheel specific developed (Figure 1) via a preliminary consensus panel with 18 attributes: 15 qualitative aimed at fully describe the smell profile of each sample, including smell intensity and persistence; 3 hedonic (frankness, aromatic richness, overall pleasantness). Each attribute was evaluated on a 0–9 scale. All ratings were digitally acquired by the Input Sensory Soft 2.0 (ISS, Centro Studi Assaggiatori, Brescia, Italy).

### 2.4. Emotional Evaluation (Explicit Method)

The emotional reaction evoked by the Piperaceae and aromatic herbs assessed was evaluated by the panelists through a questionnaire including a small selection of positive, neutral, and negative terms [26,34]. According to previous research [27], a final set of 6 emotions was proposed: fear, anger, sadness, joy/happiness, disgust, and surprise. For each sample, after completing the classic sensory evaluation panelists were asked to rate their emotions on the 0–9 scale starting from the list of the above mentioned six basic emotions. The panelists were also encouraged to freely select and indicate additional emotional descriptors if necessary.

### 2.5. Emotional Evaluation (Implicit Method)

#### 2.5.1. Instrumentation

When it comes to the implicit measurement of emotions, biomedical signals employed in the present study (ECG, GSR, EEG) were acquired in a calm, quiet, ventilated room, following a procedure already described elsewhere (see, for example [27]. In more detail, signals were acquired from all participants singularly at first for a 3′ period to capture the signal baseline, followed by their acquisition during the spices’ smelling (each spice was presented at both nostrils of the participant, at approximately 2 cm distance, for 1″, followed by 30″ interval between two consecutive compounds) and by a recovery phase, similar to the baseline, but performed after the presentation of odorous samples.

The devices employed for signal acquisition include commercial sensors: the Shimmer ECG Unit (Shimmer Research Ltd., Dublin, Ireland) for the ECG acquisition, the corresponding Shimmer3GSR+ Unit (Shimmer Research Ltd., Dublin, Ireland) for the GSR signal, and the Insight 5 Channel Wireless EEG headset (Emotiv Inc., San Francisco, CA, USA) for the EEG signal.

From a technical point of view, the ECG signal was acquired at a sampling frequency of 500 Hz in order to largely comply with the related guidelines for Heart Rate Variability (HRV) features extraction [35], whereas the GSR signal was captured at 51.2 Hz, which is one of the frequencies allowed by the Shimmer3GSR+ Unit firmware, and well beyond the sampling rates recommended (as minimum requirements) by international standards (see [36] for the related literature).

As for the Emotiv Insight EEG device, it was equipped with five EEG dry polymer sensors for brain activity detection and two CMS/DRL references (left/right mastoid process alternative). CMS (Common Mode Sense) is an active electrode and DRL (Driven Right Leg) is a passive electrode [37]. According to the 10–20 International system, the electrodes were identified as AF3, AF4, T7, T8, and Pz, corresponding to regions in the frontal lobe (frontal cortex), temporal lobe (parietal-temporal lobe) and parietal lobe (parietal-occipital cortex). The EEG signals were recorded at a sampling frequency of 128 Hz with a resolution of 16 bits and a frequency response of 0.5–43 Hz.

#### 2.5.2. Signal Processing and Features Extraction

##### ECG

The ECG trace was analyzed to extract some of the most significant features typically associated with the signal by means of a MATLAB (The MathWorks, Inc., Natick, MA, USA)-based routine. In particular, the ECG signal was first pre-processed aimed at removing artifacts, and at detecting QRS complexes, to reconstruct and correct RR series to avoid non-sinusoidal beats, thus reflecting the autonomic activity. After this procedure, both time- and frequency-domain features were extracted, including the following:

Time-domain features:

○ Heart rate (HR): the number of heartbeats recorded within a time unit. It is measured in beats per minute (bpm), and is usually associated with the sympathetic activity of the ANS.
○ Standard deviation of the normal R–R intervals (SDNN): it is an estimate of the HRV influenced by both the sympathetic and para-sympathetic branches of the ANS. It is measured in ms.
○ Root mean square of the successive differences (RMSSD): it represents the root mean square of the differences between R–R intervals close to each other. It is an estimate of the parasympathetic activity of the ANS and it is measured in ms.
○ Number of normal R–R intervals differing for more than 50 ms (NN50): it is capable of estimating the number (or the percentage) of the normal R–R intervals differing for more than 50 ms from each other. Under certain experimental conditions, as can be assumed in the present study (i.e., resting state short-term recordings), it can be referred to the parasympathetic activity of the ANS.
○ Variance of the R–R intervals (VAR): it refers to the variability of the R–R intervals.
○ SD1: standard deviation of the projection of the Poincaré plot on the perpendicular line to the identity. It estimates the short-term HRV.
○ SD2: standard deviation of the projection of the Poincaré plot on the parallel line to the identity. It estimates the long-term HRV.
○ Cardiac sympathetic index (CSI): it is obtained by the Poincaré plot and calculated as the ratio between the standard deviation of the projection of the plot on the parallel line to the identity and that on the perpendicular line (SD2/SD1). It represents a reliable sympathetic indicator.
○ Cardiac vagal index (CVI): it is obtained by the Poincaré plot and calculated as log10 (SD1 × SD2). It is assumed to be a good parasympathetic indicator.

Frequency-domain features:

○ Low frequency (LF): power spectral density of the ECG signal at low frequencies (0.04–0.15 Hz). It is usually considered to estimate the sympathetic activity of the ANS.
○ High frequency (HF): power spectral density of the ECG signal at high frequencies (0.15–0.4 Hz). It represents an estimator of both the sympathetic and parasympathetic activity of the ANS.
○ Low-to-high frequency components ratio (LF/HF): it indicates the overall balance between low and high frequency components of the ECG signal. A ratio exceeding 1 is related to sympathetic dominance, whereas for values below 1, the parasympathetic nervous system appears to be prevalent. Despite being largely used, as it relies on frequency-domain features, the LF/HF can be affected more largely by artifacts than occurring for time-domain derived features.

##### GSR

The GSR signal was analyzed by means of Ledalab, a MATLAB (The MathWorks, Inc., Natick, MA, USA)-based tool developed for processing this type of signal. With Ledalab, the GSR signal was first filtered using a first-order Butterworth low-pass filter at 5 Hz, in order to remove high frequency noise, and then administered a continuous decomposition analysis to extract both tonic and phasic phases of the signal.

Afterwards, the main features of the signal were extracted, such as the following:

Global GSR signal: composed of the sum of the tonic and phasic components of the signal.

Tonic GSR component: it refers to slow changes in the electrical signal produced by the skin. It represents the main contribution at rest and during relaxation.

Phasic GSR component: it refers to the quick stimulus-specific changes in the GSR signal, and is often termed as the skin conductance response (SCR).

##### EEG

The EEG signal was synchronized with the ECG signal by means of a MATLAB-based routine. Specifically, event markers from the ECG were carried over to EEG signal to ensure alignment with time stamps. The raw EEG signal was pre-processed using the EEG pre-processing and Transformation pipeline provided by the EMOTIVPRO Analyzer software (4.4.5.561 version) [38]. This procedure applied baseline correction, high-pass filtering at 0.5 Hz, and the Fast Fourier Transform to compute the power spectrum. The slew limit voltage was set at 100 microvolts, and the mean was used as reference value to adjust how the EEG voltage at each sensor was expressed.

The following frequency bands were defined by EMOTIVPRO for the resulting EEG power spectrum: 4–8 Hz (theta), 8–12 Hz (alpha), 12–18 Hz (low beta), 18–25 Hz (high beta), 25–32 Hz (low gamma), and 32–40 Hz (high gamma). Power in each sub-band was computed in 2 s-long windows with 75% overlap. The electrodes were grouped by brain region: frontal (AF3, AF4), temporal (T7, T8) and posterior (Pz). The epoch power of each group was averaged over the three different experimental conditions, i.e., baseline, task (odor exposure) and relaxation. That is, the spectral power of EEG epochs belonging to the same experimental conditions were averaged together. Finally, the power data were exported as a MS Excel file for the subsequent statistical analysis.

### 2.6. Statistical Analysis

Regarding the implicit assessment of emotions via wearable sensors and biomedical signals, two approaches were followed. Each subject’s response to the single stimuli was taken into account and divided into two categories according to: (i) the self-reported judgment of emotions perceived (positive vs. negative emotions), and (ii) the median value of self-reported pleasantness, expressed as a discrete value between 1 (totally unpleasant) and 9 (totally pleasant). To check for the eventual occurrence of a gaussian distribution of data, we employed the Shapiro–Wilk Test for normality. After ensuring that data deviate from normality, we have then applied non-parametrical approaches, including the Wilcoxon Signed-Rank Test for dependent variables, for statistical processing of data. Statistically significant differences were considered as being those reporting a *p*-value below 0.05.

## 3. Results and Discussion

### 3.1. Chemical Analysis of the Spontaneous Volatile Emission Profiles of All the Studied Spices

Among the 10 investigated botanicals, 5 (*Piper cubeba*, *Piper longum*, *Piper nigrum* (black p. in powder or in grains) and *Piper nigrum* (red Kampot) belonged to the Piperaceae family, while the other 5 (*Pimpinella anisum*, *Elettaria cardamomum*, *Dipterix odorata*, *Schinus molle*, and *Pimenta dioica*) belonged to five different plant families. Their spontaneous volatile emission profiles obtained by headspace solid phase micro-extraction (HS-SPME) are reported in Table 2 and Table 3. HS-SPME coupled with GC-MS is a rapid and simple method able to extract and identify compounds emitted from spices in ppm quantities and that can be used in routine analyses [39].

Except for long pepper, monoterpene hydrocarbons (MHs) dominated the Piperaceae HSs (Table 2), with relative abundances ranging from 75.3% in the whole black pepper up to 94.7% in Red Kampot peppercorn. Among MHS, however, quali-quantitative differences were evidenced among the species. Sabinene was the most abundant compound (34.6%) in Cubeb pepper HS, while it was detected with a relative content over 1% only in the whole black pepper sample. Its odor contribution is described as woody and terpenic, with a spice nuance [40]. Limonene was detected as the most abundant compound in the HSs of both the black pepper samples (41.4% and 31.5% in the whole and crushed samples, respectively), but its relative presence was also quantitatively significant in red Kampot peppercorn (25.1%) and Cubeb pepper (14.5%). This MH has a pleasant, lemon-like aroma [41]. δ-3-Carene, a MH characterized by a sweet and pungent odor [40], was the most represented compound in red Kampot peppercorn (34.8%). The spice analyzed in the present study comes from Cambodia: published research dealing with another Cambodian sample reported its essential oil (EO) analysis, which, contrary to our findings, exhibited β-caryophyllene, a sesquiterpene hydrocarbon (SH), as the main compound [42]. Limonene also accounted for up to 29.1% of the crushed black pepper HS (while it was not detected in the whole sample, thus suggesting that it is strongly retained from the matrix when it is not damaged by mechanical crushing). Among MHs, β-pinene was detected in all samples, apart from long pepper, in relative concentrations over 3%, reaching up to 13.6% in whole black pepper HS; it confers an odor described as woody, resinous, and fresh [40]. Long pepper volatile emission was, instead, dominated (71.8%) by SHs, among which β-caryophyllene exhibited the highest (22.4%) relative concentration, and was also the dominant compound in the whole HS: its aroma contribution can be described as woody and spicy [40]. Notably, long pepper was also the only sample emitting non-terpene derivatives (NTs): their presence was quantitatively relevant (19.2%) and, among them, *n*-pentadecane (9.3%) and *n*-tridecane (5.1%) were the most abundant. This predominance of SHs and NTs in the volatile emission of long pepper fruits is consistent with a published work on an Indian specimen [43].

The HS compositions of all the other studied herbs are reported in Table 3.

Consistently with previous published reports [44], phenylpropanoids (PPs) dominated the anise seeds HS emission, almost completely represented by (*E*)-anethole (91.1%). This compound has a strong odor, and it characterizes the well-known anise aroma with its sweet and licorice-like contribution [40].

Over 98% of the HS of cardamom seeds were represented by monoterpenes, mostly in their oxygenated form (86.8%). As the prevalent compound in the HS of this sample, 1,8-Cineole was detected, representing almost half (43.2%) of the total emission. This oxygenated monoterpene (OM) is commonly reported as a quantitatively relevant compound among the volatiles produced by this species [45,46], contributing to cardamom typical aroma bouquet with its herbal, minty, and eucalyptus-like odor [40].

Monoterpenes were also the main detected chemical class of volatiles in the HS of crushed pimento, with a slight predominance of their hydrocarbon form compared to the oxygenated one (39.9% and 31.9%, respectively), despite the most abundant (29.4%) compound of the total emission being 1,8-cineole, an OM. Among MHs, the most represented were δ-3-carene (9.5%), limonene (9.6%), α-pinene (6.0%), and β-pinene (5.7%). PPs represented over 20% of the total HS, with methyl eugenol as the most abundant (16.8%): this compound is characterized by a warm, spicy, cinnamon-like aroma contribution [40], and is frequently reported as the main component of the essential oil obtained from its berries [47,48].

The HS of pink peppercorn was almost completely (99.8%) composed of MHs, consistently with previously published studies on its EO composition [49,50], among which α-pinene (22.4%), sabinene (20.3%), and α-phellandrene (20.3%) were the most abundant. The woody and terpenic odor contribution of α-pinene and sabinene, thus, are enforced with the same odor contribution of α-phellandrene, whose aroma is also described as black pepper-like [40].

As already reported in the published literature [51], the HS emission of tonka beans was almost completely (95.6%) composed of coumarin, with its distinctive sweet, warm, herbaceous, and slightly spicy odor [40].

### 3.2. Sensory Analysis and Emotional Evaluation (Explicit Methods)

Spices and aromatic herbs have been a key component of the human diet since ancient times, serving as an essential source of phytochemicals for both flavor and medicinal applications. According to the U.S. Food and Drug Administration (US-FDA), spices are defined as “any aromatic vegetable substance in the whole, broken, or ground form whose significant function in food is seasoning rather than nutritional” [52]. Spices, including their extracts, tinctures, and essential oils, are widely utilized by consumers for their unique flavors and by the pharmaceutical, cosmetic, and food industries for various applications [53].

Despite the widespread use of spices and dried herbs as food seasonings across various cultures, few studies have evaluated their specific sensory profiles using trained panelists. To the best of our knowledge, no data are currently available on the correlation between volatile organic compounds (VOCs), olfactory profiles, and the emotions elicited during smell evaluation.

**Figure 2:**
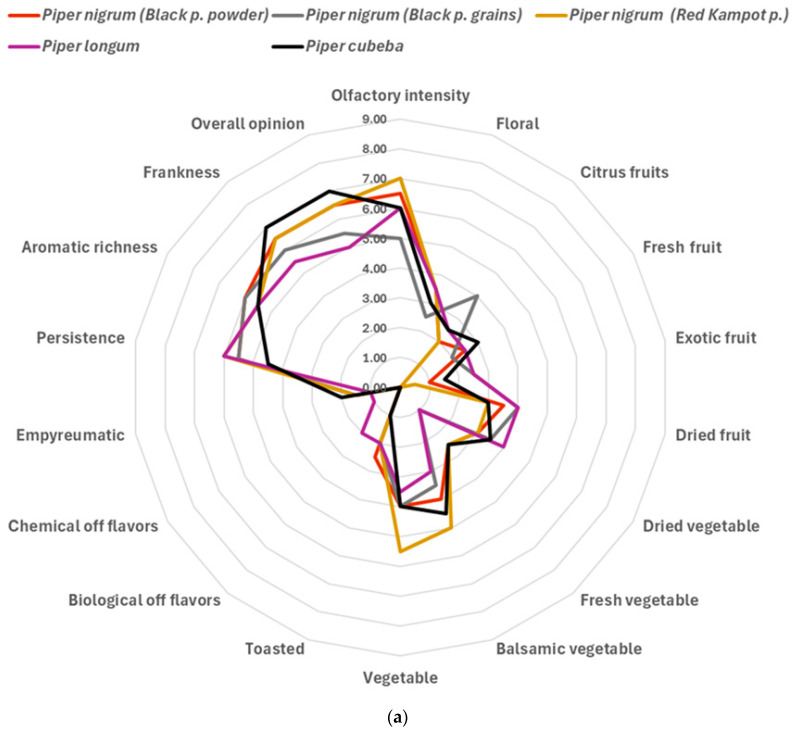

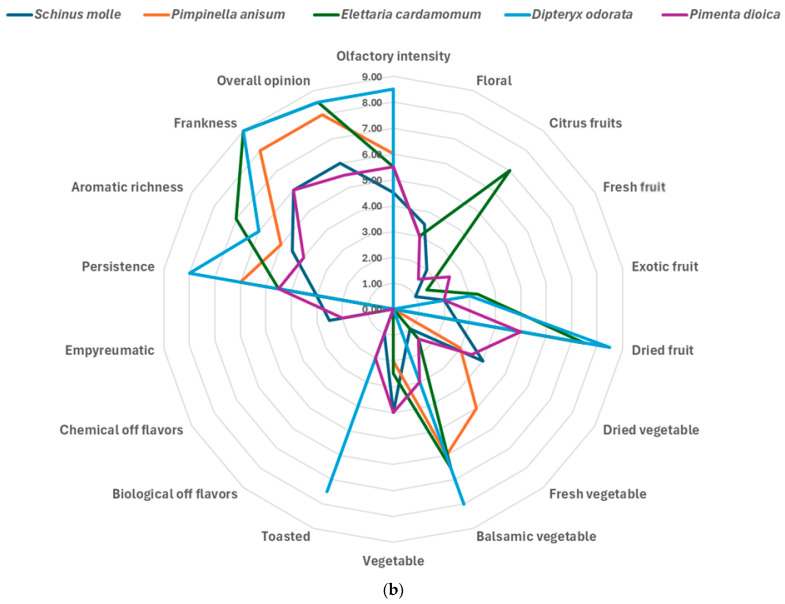
(**a**) Olfactory profile of Piperaceae. (**b**) Olfactory profile of Herbs.

In the present study, olfactory profiles of all the samples are shown in Figure 2a,b. Due to the large number of VOCs detected via GC-MS, each sample was characterized by a broad spectrum of scent nuances, ranging from vegetal to fruity notes, in accordance with their relative volatile chemical profiles. In each group (Figure 2a,b), the smell profiles of the samples differed in terms of both macro-parameters (i.e., Intensity, Frankness, and Aromatic Richness) and specific olfactory notes. As expected, the most significant differences were observed in the group of Herbs.

More in depth, among Piperaceae (Figure 2a) the best smell profile was described for *Piper cubeba*, together with *Piper nigrum* (Red Kampot) and *Piper nigrum* (Black p. in powder), while *Piper longum* showed the worst one with the lowest Overall quality mainly due to the presence of some chemical off-flavors.

Among Herbs (Figure 2b) *Schinus molle* as well as *Pimenta dioica* showed the lowest quality scores, maybe due to very low values of Smell Intensity and Persistence together with some Empyreumatic notes.

Regarding emotional evaluation, a preliminary global observation of the results obtained certainly leads us to emphasize the difficulty people face today in discussing their emotional state. Nearly all the participants, while with a predefined list of emotions to choose from, when they were asked to include even some extra emotions freely selected, they used emotions, moods, memories of specific situations, etc., in a completely interchangeable manner. This difficulty reveals a sort of emotional illiteracy, where it becomes truly challenging to express in words what one is feeling.

**Figure 3:**
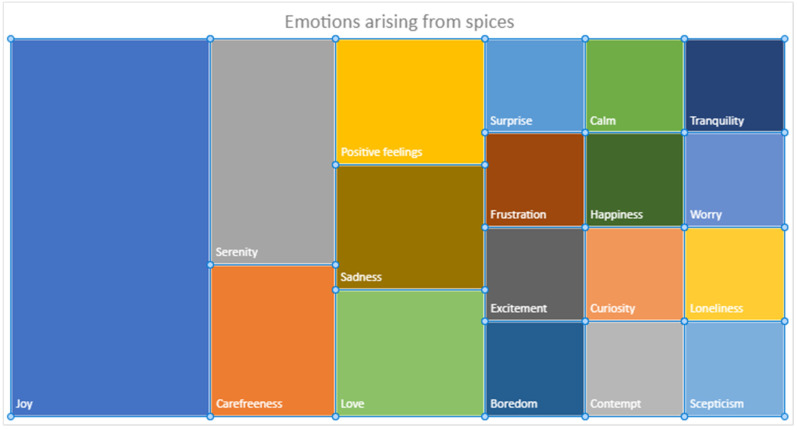
Emotions/moods whole spectrum evoked by spices.

Considering the overall responses collected during the test (Figure 3), excluding the memories of specific situations, a global analysis of the emotions/moods evoked by spices clearly reveals the broad emotional potential associated with these products. There is a predominance of positive emotions (the most represented emotion by far is joy), but there are also some references to moods related to anxiety and worry.

**Figure 4:**
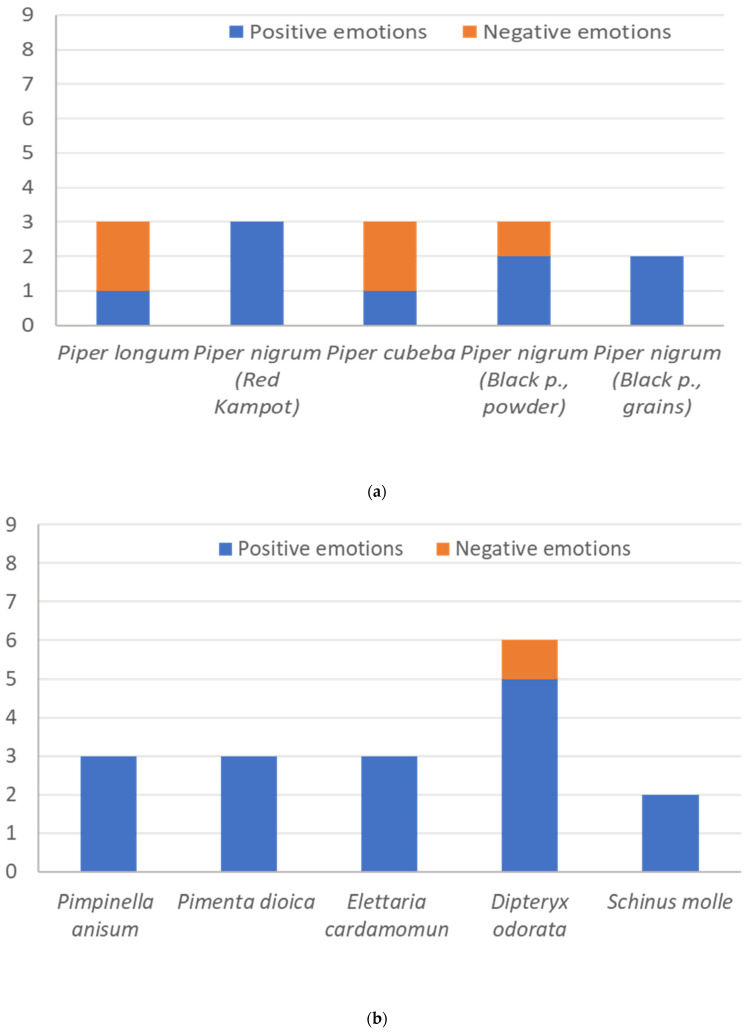
(**a**). Specific pattern of emotions of Piperaceae. (**b**) Specific pattern of emotions of herbs.

Delving into more detail and analyzing the emotional potential of individual products (Figure 4a,b), it becomes evident that there are no products with an exclusively negative connotation. On the contrary, some products can be identified as having an exclusively positive connotation, such as *Piper nigrum* L. (black p. in grain), *Piper nigrum* L. (Kampot), *Schinus molle*, *Pimenta dioica* L. and *Elettaria cardamomum*. All other products evoked a mix of positive and negative sensations.

More in depht, among the Piperaceae, *Piper cubeba* and *Piper longum* primarily evoked negative emotions. While this negative emotional response was expected for *Piper longum*, due to the presence of chemical off-flavors that reduced the overall quality of its aroma (Figure 2a), it was less expected for *Piper cubeba*. This result could be explained by the fact that, among the selected Piperaceae, *Piper cubeba* was the only one characterized by a significant presence of α-thujene in its VOCs profile (Table 2). The woody and overly green character of this volatile compound may have evoked negative emotions in some panelists, without affecting the overall mean quality rating of the aroma.

Furthermore, the slight presence of biological off-flavors detected in powdered black pepper may explain why this form elicited some negative emotions that were not observed with whole black peppercorns.

Herbs primarily evoked positive emotions (Figure 4b), with Tonka beans exhibiting the best overall emotional profile, alongside the unique negative emotion selected by the panelists. The combination of significant notes of toasted, dried fruit, and balsamic vegetables may explain the complex and varied emotional impact of this aroma.

### 3.3. Emotional Evaluation by Implicit Methods

#### 3.3.1. Relationships with Self-Reported Emotions

As stated, the first analysis was carried out to investigate around the relationship between physiological signals, acquired through wearable sensors, and self-reported emotions (Figure 4a,b) for each of the stimuli administered to the volunteers entering the present study. To do so, the single response explicitly provided in a written form by every participant to the ten spices was separately considered and divided depending on its negative or positive connotation. After this separation, the two clusters (all the negatively vs. all the positively judged emotions) were considered in terms of their detected physiological responses by each of the volunteer, and statistics (described in the dedicated Section 2) was applied to seek for a discrimination between the two clusters composed in this way.

At the *p* = 0.05 level, chosen as the cut-off value for deeming a difference as significant, no difference was reported in the variables considered. However, if we also consider the marginal significance (*p* = 0.10), the RMSSD, variable extracted from the ECG signal, was seen to be different between the two groups (*p* = 0.06), with the emotions judged as positive eliciting higher values for RMSSD than negative counterparts. In this regard, our results are suggestive of a vagally mediated response, which turned out to be more pronounced during emotionally positive stimulations [22,54].

#### 3.3.2. Relationships with Perceived Pleasantness

Afterwards, we evaluated the changes in physiological signals based on the perceived pleasantness of the single stimuli. To carry out this analysis, each participant was asked to judge every single compound administered in terms of their respective pleasantness on a 0–9 scale (the higher the more pleasant). After collecting all the responses, two clusters were identified and divided depending on the stimulus being judged as pleasant below or above the median value of all responses. After this separation, as performed with the previously explained analysis, the two clusters were considered in terms of the physiological responses of each of the volunteer, and statistics was applied to investigate eventual differences between the two clusters composed in this way.

**Figure 5:**
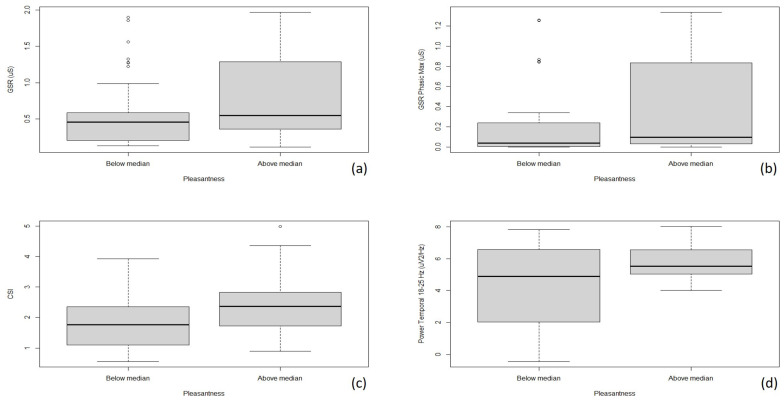
Group distribution of the mainly significant biomedical signal features depending on perceived pleasantness ((**a**) total GSR, (**b**) GSR phasic peak, (**c**) CSI, (**d**) power of the temporal region between 18 and 25 Hz).

As such, the skin conductance signals were significantly different between the two groups, with the total GSR discriminating between the them (*p* = 0.03), similarly to the peak of the phasic GSR (*p* = 0.02) and, although marginally, to the overall phasic phase (*p* = 0.07). Similarly, when it comes to the ECG-related features, CSI was significantly different between the two groups, with higher values for those reporting the odors as more pleasant (*p* = 0.01). Taken together, such results can be suggestive of a higher sympathetic arousal for those stimulation turning out to be more emotionally pleasant, which, according to our results, tend to be more exciting than less pleasant counterparts [55,56]. Finally, the EEG features were just marginally significantly different between the groups, in particular the power at the temporal level in the band 18–25 Hz (*p* = 0.09) (see Figure 5). It is worth remarking that some key structures for olfactory processing, including the limbic system, are located just nearby the temporal areas, particularly beneath the medial temporal lobe of the cerebrum, mainly in the forebrain, and are in charge for emotional and behavioral responses to external stimuli. In this regard, higher power in the 18–25 Hz band (beta waves) is suggestive of higher concentration and vigilance, which appears to be more present with more pleasant stimuli, probably also reflecting the higher emotional arousal provoked by such compounds in the volunteers enrolled [57].

## 4. Conclusions and Future Works

In the present study, we focused on a selection of aromatic herbs and Piperaceae, which are key components of nutrition in several cultures from many different regions of the globe, featuring chemical compounds capable of conveying emotions in consumers [58,59]. Until now, no specific papers dealing with a complete characterization of these kinds of botanicals from chemical, sensory and emotional perspectives have been published, highlighting the need for filling in this significant literature gap. With the current pilot investigation, we sought to contribute by merging explicit and implicit measurements on sensory analysis related to spices, also complemented by an in-depth chemical assessment related to the compounds used in the research. Interesting results emerged from the investigation, with the full characterization of chemical composition of the ten botanicals considered, and unprecedented evidence towards psychophysiological responses to olfactory stimuli conveyed by such iconic edible compounds. The pilot nature of the current investigation prevents us from providing the scientific community with fully generalizable evidence for such mechanisms. However, future research could apply the same principles to a broader cohort, well-balanced across ages and genders, fully characterized from a sensory perspective, and possibly focusing more on the investigation around the relationship between physiological measurements and pleasantness, rather than with the positive or negative self-judgment of emotions, as derived from the results obtained.

According to perceptual theories, emotions provide valuable information about our physiological and psychological reactions to external situations. At the same time, the signals generated by our body’s responses to stimuli, such as the taste of food, can be identified and quantified.

In both generic tasting experiences and structured panel tests, the evaluation of a product stems from the interplay between the organoleptic properties of the food and the contextual stimuli. Together, these factors create a holistic sensory experience. The subsequent decision-making process is based on the interpretation of these combined results, further influenced by external factors unrelated to the food itself, such as social environment, memories, emotions, and cultural background. These elements collectively shape the perceived quality of the food. And some of those characteristics can be investigated using wearable devices for monitoring physiological parameters, like those derived from biomedical signals like ECG, skin conductance, EEG, and so forth. Additionally, the pleasantness of the experience can be influenced by prior actions designed to create expectations about the product’s quality, possibly conveyed also by means of Virtual- (VR), Augmented- (AR) and Mixed-Reality (XR) scenarios designed ad hoc, possibly flowing into consumer science and neuromarketing.

While the essential role of emotions in sensory evaluation and their impact on consumer preferences and purchasing decisions is widely acknowledged, it remains unclear how emotions should be systematically used to define food preferences or assess food quality.

Overall, the findings of this pilot study, along with existing results for other food categories beyond spices and herbs, suggest the potential to develop new consumer tests that incorporate both explicit and implicit methods to measure the emotional responses elicited by foods, herbs, and spices. Such an approach could help guide consumer choices and optimize the overall food experience.

## Figures and Tables

**Table 1:** Botanicals used for experimental runs.

Botanical Name	Family	Native	Common Name in English, French, Spanish, German and Italian	Organ or Part Used
Herbs				
*Pimpinella anisum* L.	*Apiaceae*	Southwest Asia, Greece, Egypt, and India	(eng) anise, (fra) anis, (esp) anís, (deu) anis, (ita) anice	Aniseed fruit
*Elettaria cardamomum* (L.)	*Zingiberaceae*	Southern India	(eng) cardamom, (fra) cardamome, (esp) cardamomo, (deu) kardamom, (ita) cardamomo	Seeds
*Dipteryx odorata* (Aubl.) Forsyth f.	*Fabaceae*	Central and South America	(eng) tonka bean, (fra) fève tonka, (esp) haba tonka, (deu) tonkabohne, (ita) fava tonka	Seeds and fruit
*Schinus molle* L.	*Anacardiaceae*	Brazil	(eng) pink pepper or false pepper, (fra) faux poivrier, (esp) pimienta de brasil o pimienta rosada, (deu) rosa pfeffer, (ita) pepe rosa	Pepper fruit
*Pimenta dioica* (L.) Merr.	*Myrtaceae*	West Indies and Central America especially Jamaica and Cuba	(eng) jamaica pepper or allspice, (fra) poivre de la jamaïque, (esp) pimienta de jamaica, (deu) jamaika pfeffer, (ita) pepe garofanato	Berries and leaf
Piperaceae				
*Piper longum* L.	*Piperaceae*	India	(eng) long pepper, (fra) poivre long, (esp) pimienta larga, (deu) langer pfeffer, (ita) pepe lungo	Pepper fruit and dried leaf
*Piper cubeba* L.f.	*Piperaceae*	India	(eng) cubeb pepper, (fra) poivre cubèbe, (esp) pimienta cubeba, (deu) kubeben pfeffer, (ita) pepe cubebe	Pepper fruit
*Piper nigrum* L. (black p. in powder)	*Piperaceae*	India	(eng) black pepper, (fra) poivre noir, (esp) pimienta negra, (deu) schwarzer pfeffer, (ita) pepe nero	Peppercorn
*Piper nigrum* L. (black p. in grains)	*Piperaceae*	India	(eng) black pepper, (fra) poivre noir, (esp) pimienta negra, (deu) schwarzer pfeffer, (ita) pepe nero	Peppercorn
*Piper nigrum* L.	*Piperaceae*	Cambodia	(eng) Kampot pepper, (fra) poivre de Kampot, (esp) pimienta roja Kampot, (deu) Kampot-pfeffer, (ita) pepe rosso di Kampot	Peppercorn

**Table 2:** Complete compositions of the spontaneously emitted volatiles in the headspace of all the studied *Piperaceae* spp.

Compounds	l.r.i. ^a^	Relative Abundance (%) ± SD ^b^				
		*Piper cubeba*	*Piper nigrum* (Black p. in Powder)	*Piper nigrum* (Black p. in Grains)	*Piper Longum*	*Piper nigrum* (Red Kampot)
α-thujene	931	10.6 ± 0.28	- ^c^	1.2 ± 0.04	-	-
α-pinene	941	7.3 ± 0.25	1.0 ± 0.21	4.9 ± 0.06	1.0 ± 0.16	4.0 ± 0.39
sabinene	976	34.6 ± 0.69	0.2 ± 0.02	11.0 ± 0.13	0.2 ± 0.04	0.2 ± 0.02
β-pinene	982	3.6 ± 1.17	5.8 ± 0.56	13.6 ± 0.01	0.9 ± 0.23	11.3 ± 0.47
myrcene	993	1.6 ± 0.20	3.7 ± 0.08	2.6 ± 0.04	0.1 ± 0.01	4.5 ± 0.20
α-phellandrene	1005	1.5 ± 0.03	7.4 ± 0.18	0.1 ± 0.17	-	9.3 ± 0.31
δ-3-carene	1011	0.5 ± 0.04	29.1 ± 0.77	-	0.4 ± 0.08	34.8 ± 0.08
α-terpinene	1018	0.7 ± 0.04	0.6 ± 0.66	0.1 ± 0.11	-	0.9 ± 0.36
*o*-cymene	1024	-	0.3 ± 0.21	-	-	-
*p*-cymene	1027	1.6 ± 0.06	2.2 ± 0.17	0.1 ± 0.19	0.1 ± 0.00	1.9 ± 0.09
limonene	1032	14.5 ± 1.17	31.5 ± 0.21	41.4 ± 0.38	1.5 ± 0.18	25.1 ± 0.24
1,8-cineole	1034	0.9 ± 0.47	-	-	0.3 ± 0.03	-
(*Z*)-β-ocimene	1042	-	-	-	0.3 ± 0.06	-
(*E*)-β-ocimene	1052	0.1 ± 0.01	-	-	0.3 ± 0.02	-
γ-terpinene	1062	1.2 ± 0.00	0.6 ± 0.01	0.1 ± 0.20	-	0.5 ± 0.03
acetophenone	1068	-	-	-	1.1 ± 0.79	-
*cis*-sabinene hydrate	1070	0.4 ± 0.04	-	0.1 ± 0.11	-	-
terpinolene	1088	0.3 ± 0.02	3.1 ± 0.10	0.1 ± 0.17	-	2.1 ± 0.01
linalool	1101	2.9 ± 0.35	0.2 ± 0.03	-	1.8 ± 0.18	0.1 ± 0.07
4-terpineol	1178	0.2 ± 0.00	-	-	-	-
(*E*)-anethole	1283	-	-	0.2 ± 0.00	0.7 ± 0.03	-
*n*-tridecane	1300	-	-	-	5.1 ± 0.80	-
piperonal	1333	-	-	-	0.2 ± 0.04	-
δ-elemene	1340	0.6 ± 0.00	1.1 ± 0.18	-	0.3 ± 0.06	0.1 ± 0.07
α-cubebene	1350	3.3 ± 0.04	0.1 ± 0.02	0.2 ± 0.01	0.4 ± 0.11	-
α-ylangene	1372	-	-	-	0.1 ± 0.01	-
α-copaene	1376	3.1 ± 0.02	2.1 ± 0.42	4.7 ± 0.05	1.2 ± 0.01	-
β-cubebene	1390	3.9 ± 0.08	-	0.2 ± 0.00	0.3 ± 0.06	-
β-elemene	1392	0.4 ± 0.04	0.1 ± 0.01	0.1 ± 0.00	3.6 ± 0.24	0.1 ± 0.08
α-gurjunene	1410	0.2 ± 0.01	-	-	-	-
*cis-*α-bergamotene	1416	-	-	-	1.1 ± 0.08	-
β-caryophyllene	1420	1.7 ± 0.06	10.3 ± 1.99	17.7 ± 0.21	22.4 ± 0.45	4.8 ± 0.54
β-copaene	1429	0.1 ± 0.08	-	-	0.5 ± 0.00	-
*trans*-α-bergamotene	1438	-	0.3 ± 0.15	-	1.6 ± 0.04	-
α-humulene	1456	0.7 ± 0.04	0.3 ± 0.07	0.5 ± 0.01	9.6 ± 0.25	0.2 ± 0.02
(*E*)-β-farnesene	1460	-	-	-	1.3 ± 0.21	-
*allo*aromadendrene	1461	0.5 ± 0.01	-	-	-	-
γ-muurolene	1477	0.5 ± 0.01	-	-	0.5 ± 0.05	-
germacrene D	1478	0.6 ± 0.04	-	-	9.1 ± 0.01	-
*ar*-curcumene	1483	-	-	-	1.9 ± 0.05	-
β-selinene	1485	0.1 ± 0.07	-	-	3.1 ± 0.24	0.1 ± 0.09
1-pentadecene	1488	-	-	-	3.4 ± 0.16	-
α-selinene	1494	0.4 ± 0.01	-	-	1.6 ± 0.01	-
bicyclogermacrene	1496	0.3 ± 0.05	-	-	-	-
α-zingiberene	1496	-	-	-	1.6 ± 0.71	-
α-muurolene	1498	0.2 ± 0.01	-	-	-	-
*n*-pentadecane	1500	-	-	-	9.3 ± 0.63	-
(Z)-α-bisabolene	1504	-	-	-	1.1 ± 0.08	-
β-bisabolene	1509	-	-	0.4 ± 0.01	5.8 ± 0.43	-
cubebol	1516	0.7 ± 0.04	-	-	-	-
7-*epi*-α-selinene	1517	-	-	-	2.0 ± 0.18	-
δ-cadinene	1525	0.3 ± 0.01	0.1 ± 0.09	0.3 ± 0.01	-	-
β-sesquiphellandrene	1525	-	-	-	0.7 ± 0.15	-
(*E*)-γ-bisabolene	1535	-	-	-	0.5 ± 0.08	-
germacrene B	1554	-	-	-	1.4 ± 0.15	-
caryophyllene oxide	1581	-	-	-	0.5 ± 0.01	-
humulene epoxide II	1608	-	-	-	0.1 ± 0.11	-
Monoterpene hydrocarbons		78.1 ± 0.08	85.4 ± 2.96	75.3 ± 0.18	4.8 ± 0.52	94.7 ± 0.81
Oxygenated monoterpenes		4.4 ± 0.08	0.2 ± 0.03	0.1 ± 0.11	2.0 ± 0.16	0.1 ± 0.07
Sesquiterpene hydrocarbons		16.8 ± 0.03	14.4 ± 2.93	24.2 ± 0.29	71.8 ± 1.9	5.1 ± 0.81
Oxygenated sesquiterpenes		0.7 ± 0.04	-	-	0.6 ± 0.09	-
Phenylpropanoids		-	-	0.2 ± 0.00	0.7 ± 0.03	-
Non-terpene derivatives		-	-	-	19.2 ± 0.85	-
Total identified (%):		100 ± 0.01	100 ± 0.00	99.7 ± 0.01	99.1 ± 0.26	99.8 ± 0.06

^a^ Linear retention index on a HP5-MS capillary column; ^b^ Detection threshold: 0.1%; ^c^ Not detected.

**Table 3:** Complete compositions of the spontaneously emitted volatiles in the headspace of anide seeds, cardamom seeds, crushed pimento fruits, pink peppercorn fruits, and tonka beans.

Compounds	l.r.i. ^a^	Relative Abundance (%) ± SD ^b^				
		*Pimpinella anisum* (Anise)	*Elettaria cardamomum* (Cardamom)	*Pimenta dioica* (Jamaica Pepper)	*Schinus molle* (Pink Pepper)	*Dipteryx odorata* (Tonka Bean)
butyrolactone	918	- ^c^	-	-	-	0.6 ± 0.08
α-thujene	931	-	0.4 ± 0.09	1.1 ± 0.05	1.0 ± 0.23	-
α-pinene	941	-	0.8 ± 0.16	6.0 ± 0.52	22.4 ± 4.11	-
camphene	955	-	-	0.2 ± 0.06	1.0 ± 0.37	-
benzaldehyde	959	0.2 ± 0.05	-	-	-	-
sabinene	976	0.1 ± 0.10	3.4 ± 0.33	1.0 ± 0.08	20.3 ± 2.32	-
β-pinene	982	0.1 ± 0.10	0.3 ± 0.02	5.7 ± 0.55	2.0 ± 0.54	-
myrcene	993	-	2.2 ± 0.01	2.8 ± 0.04	2.6 ± 0.31	-
α-phellandrene	1005	-	-	0.5 ± 0.01	20.3 ± 3.37	-
δ-3-carene	1011	0.2 ± 0.11	-	9.5 ± 0.64	8.0 ± 8.10	-
α-terpinene	1018	-	0.1 ± 0.11	-	0.8 ± 0.72	-
*p*-cymene	1027	0.1 ± 0.06	0.3 ± 0.04	3.1 ± 0.25	4.8 ± 1.96	-
limonene	1032	1.7 ± 1.15	4.1 ± 0.59	9.3 ± 0.13	15.6 ± 3.4	0.2 ± 0.10
1,8-cineole	1034	0.3 ± 0.16	43.2 ± 3.13	29.4 ± 1.13	-	-
(*E*)-β-ocimene	1052	-	0.1 ± 0.12	-	-	-
γ-terpinene	1062	0.2 ± 0.06	0.4 ± 0.10	0.5 ± 0.01	0.4 ± 0.30	-
*cis*-sabinene hydrate	1070	-	0.7 ± 0.04	-	-	-
1-octanol	1071	-	-	0.3 ± 0.01	-	-
terpinolene	1088	-	0.3 ± 0.16	-	0.6 ± 0.01	-
*p*-cymenene	1089	-	-	0.4 ± 0.08	-	-
linalool	1101	0.1 ± 0.02	4.8 ± 0.36	1.0 ± 0.06	-	0.1 ± 0.04
nonanal	1104	0.5 ± 0.49	-	-	-	0.2 ± 0.14
4-terpineol	1178	-	0.6 ± 0.01	1.0 ± 0.01	-	-
α-terpineol	1189	-	1.6 ± 0.16	0.5 ± 0.02	-	-
methyl chavicol	1197	2.1 ± 0.08	-	0.9 ± 0.03	-	0.3 ± 0.08
decanal	1204	0.3 ± 0.17	-	-	-	-
*p*-anisaldehyde	1256	0.7 ± 0.09	-	-	-	-
linalyl acetate	1259	-	5.1 ± 0.25	-	-	-
(*E*)-anethole	1283	91.1 ± 2.37	0.2 ± 0.01	-	-	2.1 ± 0.42
carvacrol	1298	-	-	-	-	0.2 ± 0.02
α-terpinyl acetate	1352	-	30.7 ± 1.77	-	-	-
eugenol	1358	-	-	3.8 ± 0.91	-	-
hydrocoumarin	1376	-	-	-	-	0.6 ± 0.06
α-copaene	1376	-	-	1.5 ± 0.07	-	-
geranyl acetate	1385	-	0.1 ± 0.14	-	-	-
β-elemene	1392	-	-	0.5 ± 0.06	-	-
methyl eugenol	1403	-	-	16.8 ± 1.66	-	-
β-caryophyllene	1420	-	-	3.2 ± 0.21	-	0.1 ± 0.08
coumarin	1432	-	-	-	-	95.6 ± 1.02
α-himachalene	1448	0.2 ± 0.00	-	-	-	-
α-humulene	1456	-	-	0.2 ± 0.03	-	-
germacrene D	1478	-	-	-	0.2 ± 0.04	-
β-chamigrene	1485	2.1 ± 0.08	-	-	-	-
caryophyllene oxide	1581	-	-	0.8 ± 0.11	-	-
Monoterpene hydrocarbons		2.3 ± 1.57	12.4 ± 0.63	39.9 ± 1.99	99.8 ± 0.04	0.2 ± 0.10
Oxygenated monoterpenes		0.5 ± 0.18	86.8 ± 0.40	31.9 ± 1.05	-	0.4 ± 0.06
Sesquiterpene hydrocarbons		2.3 ± 0.08	-	5.5 ± 0.37	0.2 ± 0.04	0.1 ± 0.08
Oxygenated sesquiterpenes		-	-	0.8 ± 0.11	-	-
Phenylpropanoids		93.2 ± 2.28	0.2 ± 0.01	21.4 ± 2.54	-	2.4 ± 0.50
Non-terpene derivatives		1.6 ± 0.62	-	0.3 ± 0.01	-	97.0 ± 0.74
Total identified (%):		100 ± 0.00	99.3 ± 0.25	99.8 ± 0.01	100 ± 0.01	100 ± 0.00

^a^ Linear retention index on a HP5-MS capillary column; ^b^ Detection threshold: 0.1%; ^c^ Not detected.

## Data Availability

The original contributions presented in the study are included in the article, further inquiries can be directed to the corresponding author.

## References

[1] Szűcs V., Szabó E., Lakner Z., Székács A. (2018). National seasoning practices and factors affecting the herb and spice consumption habits in Europe. Food Control.

[2] Siddiq M., Uebersax M.A. (2018). Handbook of Vegetables and Vegetable Processing.

[3] Vázquez-Fresno R., Rosana A.R.R., Sajed T., Onookome-Okome T., Wishart N.A., Wishart D.S. (2019). Herbs and spices-biomarkers of intake based on human intervention studies—A systematic review. Genes Nutr..

[4] Shan B., Cai Y.Z., Sun M., Corke H. (2005). Antioxidant capacity of 26 spice extracts and characterization of their phenolic constituents. J. Agric. Food Chem..

[5] Ceto X., Sarma M., Valle M. (2024). Analysis of spices & herbs and its phenolic content by means of an electronic tongue. LWT-Food Sci. Technol..

[6] Cuvelier M.E., Berset C., Richard H. (1994). Antioxidant constituents in sage (*Salvia officinalis*). J. Agric. Food Chem..

[7] Pizzale L., Bortolomeazzi R., Vichi S., Uberegger E., Conte L.S. (2002). Antioxidant activity of sage (*Salvia officinalis* and *S. fruticosa*) and oregano (*Origanum onites* and *O. indercedens*) extracts related to their phenolic compound content. J. Sci. Food Agric..

[8] Zheng W., Wang S.Y. (2001). Antioxidant activity and phenolic compounds in selected herbs. J. Agric. Food Chem..

[9] Moss R., Fisher C., Gorman M., Knowles S., LeBlanc J., Ritchie C., Schindell K., Ettinger L., McSweeney M.B. (2023). Effect of piperine on saltiness perception. Foods.

[10] Moss R., LeBlanc J., Ritchie C., Gorman M., Ettinger L., McSweeney M.B. (2023). Effect of white pepper addition on the sensory perception of sodium-reduced soup with an emphasis on saltiness perception. J. Sens. Stud..

[11] McNamara F.N., Randall A., Gunthorpe M.J. (2005). Effects of piperine, the pungent component of black pepper, at the human vanilloid receptor (TRPV1). Br. J. Pharmacol..

[12] Kilcast D., Angus F. (2014). Reducing Salt in Foods.

[13] Sasikumar B., Swetha V.P., Parvathy V.A., Sheeja T.E. (2016). 22—Advances in Adulteration and Authenticity Testing of Herbs and Spices. Advances in Food Authenticity Testing.

[14] Kunová S., Taglieri I., Hašcík P., Ben Hsouna A., Mnif W., Venturi F., Kačániová M. (2023). Dried Herbs as an Easy-to-Use and Cost-Effective Alternative to Essential Oils to Extend the Shelf Life of Sheep Lump Cheese. Foods.

[15] Kačániová M., Čmiková N., Ban Z., Garzoli S., Elizondo-Luevano J.H., Ben Hsouna A., Ben Saad R., Bianchi A., Venturi F., Kluz M.I. (2024). Enhancing the Shelf Life of Sous-Vide Red Deer Meat with Piper nigrum Essential Oil: A Study on Antimicrobial Efficacy against Listeria monocytogenes. Molecules.

[16] Djebbi T., Ascrizzi R., Bedini S., Farina P., Sanmartin C., Jouda Mediouni B.J., Bozzini M.F., Flamini G., Conti B. (2024). Physicochemical and repellent properties of chitosan films loaded with essential oils for producing an active packaging effective against the food pest *Sitophilus oryzae*. J. Stored Prod. Res..

[17] Embuscado M.E. (2015). Spices and herbs: Natural sources of antioxidants—A mini review. J. Funct. Foods.

[18] Nguyen L., Duong L.T., Mentreddy R.S. (2019). The US import demand for spices and herbs by differentiated sources. J. Appl. Res. Med. Aromat. Plants.

[19] Croy I., Olgun S., Joraschky P. (2011). Basic emotions elicited by odors and pictures. Emotion.

[20] Tonacci A., Taglieri I., Sanmartin C., Billeci L., Crifaci G., Ferroni G., Braceschi G.P., Odello L., Venturi F. (2023). Taste the emotions: Pilot for a novel, sensors-based approach to emotional analysis during coffee tasting. J. Sci. Food Agric..

[21] Izard C.E. (2009). Emotion theory and research: Highlights, unanswered questions, and emerging issues. Annu. Rev. Psychol..

[22] Tonacci A., Billeci L., Di Mambro I., Marangoni R., Sanmartin C., Venturi F. (2021). Wearable Sensors for Assessing the Role of Olfactory Training on the Autonomic Response to Olfactory Stimulation. Sensors.

[23] Brouwer A.M., Hogervorst M.A., Grootjen M., van Erp J.B.F., Zandstra E.H. (2017). Neurophysiological responses during cooking food associated with different emotions. Food Qual. Prefer..

[24] De Luca R., Botelho D. (2021). The unconscious perception of smells as a driver of consumer responses: A framework integrating the emotion-cognition approach to scent marketing. Acad. Mark. Sci..

[25] Meiselman H.L. (2015). A review of the current state of emotion research in product development. Food Res. Int..

[26] Billeci L., Sanmartin C., Tonacci A., Taglieri I., Ferroni G., Marangoni R., Venturi F. Wearable sensors to measure the influence of sonic seasoning on wine consumers in a live context: A preliminary proof-of-concept study. J. Sci. Food Agric..

[27] Tonacci A., Scalzini G., Díaz-Guerrero P., Sanmartin C., Taglieri I., Ferroni G., Flamini G., Odello L., Billeci L., Venturi F. (2024). Chemosensory analysis of emotional wines: Merging of explicit and implicit methods to measure emotions aroused by red wines. Food Res. Int..

[28] National Institute of Standards and Technology, NIST (2014). NIST/EPA/NIH Mass Spectral Library, NIST Standard Reference Database Number 69.

[29] Adams R.P. (2007). Identification of Essential Oil Components by Gas Chromatography/Mass Spectroscopy.

[30] Billeci L., Sanmartin C., Tonacci A., Taglieri I., Bachi L., Ferroni G., Braceschi G.P., Odello L., Venturi F. (2023). Wearable sensors to evaluate autonomic response to olfactory stimulation: The influence of short intensive sensory training. Biosensors.

[31] Parichanon P., Ascrizzi R., Tani C., Sanmartin C., Taglieri I., Macaluso M., Flamini G., Pieracci Y., Venturi F., Conti B. (2023). The protective combined effect of chitosan and essential oil coatings on cheese and cured meat against the oviposition of Piophila casei. Food Bioscence.

[32] Rossi F. (2001). Assessing sensory panelist performance using repeatability and reproducibility measures. Food Qual. Prefer..

[33] (2010). Sensory Analysis—General Guidance for the Design of Test Rooms.

[34] Ferrarini R., Carbognin C., Casarotti E.M., Nicolis E., Nencini A., Meneghini A.M. (2010). The emotional response to wine consumption. Food Qual. Prefer..

[35] Task Force of the European Society of Cardiology and the North American Society of Pacing and Electrophysiology (1996). Heart rate variability: Standards of measurement, physiological interpretation and clinical use. Circulation.

[36] Boucsein W. (2012). Electrodermal activity. Techniques in Psychophysiology.

[37] (2019). Emotiv: Emotiv Insight Brainwear 5 Channel Wireless EEG Headset. https://www.emotiv.com/insight/.

[38] (2019). Emotiv: Software. https://www.emotiv.com.

[39] Diaz-Maroto M.C., Perez-Coello M.S., Cabezucto M.D. (2002). Headspace Solid-Phase Microextraction Analysis of Volatile Components of Spices Key Words Gas chromatography-mass spectrometry Solid-phase microextraction Volatile compounds in spices Basil, oregano, and bay leaves. Chromatographia.

[40] The Good Scents Company (TGSC) Information System. https://www.thegoodscentscompany.com/index.html.

[41] Burdock G.A. (2010). Fenaroli’s Handbook of Flavor Ingredients.

[42] Katerina V., Klara U., Samnang N., Ladislav K. (2023). Chemical Composition of Essential Oils and Supercritical Carbon Dioxide Extracts from Amomum kravanh, Citrus hystrix and Piper nigrum ‘Kampot’. Molecules.

[43] Dash M., Singh S., Sahoo B.C., Sahoo S., Sahoo R.K., Nayak S., Kar B. (2021). Potential role of Indian long pepper (*Piper longum* L.) volatiles against free radicals and multidrug resistant isolates. Nat. Prod. Res..

[44] Elmassry M.M., Kormod L., Labib R.M., Farag M.A. (2018). Metabolome Based Volatiles Mapping of Roasted Umbelliferous Fruits Aroma via HS-SPME GC/MS and Peroxide Levels Analyses. J. Chromatogr. B.

[45] Noumi E., Snoussi M., Alreshidi M.M., Rekha P.D., Saptami K., Caputo L., De Martino L., Souza L.F., Msaada K., Mancini E. (2018). Chemical and Biological Evaluation of Essential Oils from Cardamom Species. Molecules.

[46] Morsy N.F.S. (2015). A short extraction time of high quality hydrodistilled cardamom (*Elettaria cardamomum* L. Maton) essential oil using ultrasound as a pretreatment. Ind. Crops Prod..

[47] Morsy N.F.S., Hammad K.S.M. (2018). Volatile Constituents, Radical Scavenging and Cytotoxic Activities of Mexican allspice (*Pimenta dioica* L. Merrill) Berries Essential Oil. J. Essent. Oil Bear. Plants.

[48] García-Fajardo J., Martínez-Sosa M., Estarrón-Espinosa M., Vilarem G., Gaset A., de Santos J.M. (1997). Comparative Study of the Oil and Supercritical CO_2_ Extract of Mexican Pimento (*Pimenta dioica* Merrill). J. Essent. Oil Res..

[49] Bendaoud H., Romdhane M., Souchard J.P., Cazaux S., Bouajila J. (2010). Chemical composition and anticancer and antioxidant activities of *Schinus molle* L. and *Schinus terebinthifolius* Raddi berries essential oils. J. Food Sci..

[50] Martins M.d.R., Arantes S., Candeias F., Tinoco M.T., Cruz-Morais J. (2014). Antioxidant, antimicrobial and toxicological properties of *Schinus molle* L. essential oils. J. Ethnopharmacol..

[51] Bajer T., Surmová S., Eisner A., Ventura K., Bajerová P. (2018). Use of simultaneous distillation-extraction, supercritical fluid extraction and solid-phase microextraction for characterisation of the volatile profile of *Dipteryx* odorata (Aubl.) willd. Ind. Crop. Prod..

[52] U.S. FDA (2017). CFR—Code of Federal Regulations Title 21—Food and Drugs Chapter I—Food and Drug Administration Department of Health and Human Services Subchapter B—Food for Human Consumption. https://www.accessdata.fda.gov/scripts/cdrh/cfdocs/cfcfr/CFRSearch.cfm?CFRPart=101&showFR=1&subpartNode=21:2.0.1.1.2.2.

[53] Modupalli N., Naik M., Sunil C.K., Natarajan V. (2021). Emerging non-destructive methods for quality and safety monitoring of spices. Trends Food Sci. Technol..

[54] Shaffer F., Ginsberg J.P. (2017). An Overview of Heart Rate Variability Metrics and Norms. Front Public Health.

[55] Straszewski T., Siegel J.T. (2020). Differential Effects of High- and Low-Arousal Positive Emotions on Help-Seeking for Depression. Appl. Psychol. Health Well Being.

[56] Cui X., Tian Y., Zhang L., Chen Y., Bai Y., Li D., Liu J., Gable P., Yin H. (2023). The role of valence, arousal, stimulus type, and temporal paradigm in the effect of emotion on time perception: A meta-analysis. Psychon. Bull. Rev..

[57] Martin C., Ravel N. (2014). Beta and gamma oscillatory activities associated with olfactory memory tasks: Different rhythms for different functional networks?. Front. Behav. Neurosci..

[58] Chrea C., Grandjean D., Delplanque S., Cayeux I., Le Calvee B., Aymard L., Velazco M., Sander D., Scherer K.R. (2009). Mapping the semantic space for the subjective experience of emotional responses to odors. Chem. Senses.

[59] Bell B., Adhikari K., Chambers E., Alavi S., King S., Haub M. (2017). Spices in a product affect emotions: A study with an extruded snack product. Foods.

